# A Timely Suggestion

**Published:** 1914-05

**Authors:** 


### A Timely Suggestion.

Commenting on the above, one is struck with the forcefulness of the arguments used for race betterment. The proposition is strictly scientific and logical and is exceedingly good as far as it goes. But we must remember that the germ-cell lies in the organs of generation and not the brain.

Educate the brain, yes. Improve its mental horizon, certainly. Increase the individual happiness and capacity, in every way possible. But such labor of love and humanity is "interred with the recipient's bones." The child's character does not begin where its father's left off, but must be built entirely new on the possibilities and limitations heredity has given him. In other words, he is a "chip off the old block."

New characters can only be formed by breeding. The mentally benefited child, improved by his environments, still has the old germ of life to transmit to his offspring. Thus the benefit is for one generation and not necessarily for posterity, unless the laws of human breeding be taken into account also. An improved intelligence will add greatly to the judgment of the individual and he may therefore exercise self-restraint in the question of sexual selection. In that way Dr. Smith's very scholarly advise may be of ultimate benefit to the race.

